# Childhood trauma, HPA axis activity and antidepressant response in patients with depression

**DOI:** 10.1016/j.bbi.2019.11.024

**Published:** 2020-07

**Authors:** Naghmeh Nikkheslat, Anna P. McLaughlin, Caitlin Hastings, Zuzanna Zajkowska, Maria A. Nettis, Nicole Mariani, Daniela Enache, Giulia Lombardo, Linda Pointon, Philip J. Cowen, Jonathan Cavanagh, Neil A. Harrison, Edward T. Bullmore, NIMA Consortium, Carmine M. Pariante, Valeria Mondelli

**Affiliations:** a Department of Psychological Medicine, Institute of Psychiatry, Psychology & Neuroscience, Kings College London, UK; b National Institute for Health Research (NIHR) Mental Health Biomedical Research Centre at South London and Maudsley NHS Foundation Trust and King’s College London, UK; c University Department of Psychiatry, Warneford Hospital, Oxford, UK; d Mental Health and Wellbeing, Sackler Institute, Neurology Block, Queen Elizabeth University Hospital, Glasgow, UK; e Department of Neuroscience, Brighton & Sussex Medical School, University of Sussex, Brighton, UK; f Department of Psychiatry, Behavioural and Clinical Neurosciences Institute, University of Cambridge, UK; g GlaxoSmithKline R&D, Stevenage UK, Cambridgeshire & Peterborough NHS Foundation Trust, Cambridge UK

**Keywords:** Major depressive disorders, Childhood trauma, HPA axis hyperactivity, Cortisol response, Inflammation, Glucocorticoid resistance, Treatment resistant depression

## Abstract

Childhood trauma is among the most potent contributing risk factors for depression and is associated with poor treatment response. Hypothalamic-pituitary-adrenal (HPA) axis abnormalities have been linked to both childhood trauma and depression, but the underlying mechanisms are poorly understood. The present study aimed to investigate the link between childhood trauma, HPA axis activity and antidepressant response in patients with depression. As part of the Wellcome Trust NIMA consortium, 163 depressed patients and 55 healthy volunteers were included in this study. Adult patients meeting Structured Clinical Interview for Diagnostic and Statistical Manual Version-5 criteria for major depression were categorised into subgroups of treatment responder (n = 42), treatment non-responder (n = 80) and untreated depressed (n = 41) based on current depressive symptom severity measured by the 17-item Hamilton Rating Scale for Depression and exposure to antidepressant medications established by Antidepressant Treatment Response Questionnaire. Childhood Trauma Questionnaire was obtained. Baseline serum C-reactive protein was measured using turbidimetric detection. Salivary cortisol was analyzed at multiple time points during the day using the ELISA technique. Glucocorticoid resistance was defined as the coexistence of hypercortisolemia and inflammation. Our results show that treatment non-responder patients had higher exposure to childhood trauma than responders. No specific HPA axis abnormalities were found in treatment non-responder depressed patients. Untreated depressed showed increased diurnal cortisol levels compared with patients on antidepressant medication, and higher prevalence of glucocorticoid resistance than medicated patients and controls. The severity of childhood trauma was associated with increased diurnal cortisol levels only in individuals with glucocorticoid resistance. Therefore, our findings suggest that the severity of childhood trauma experience contributes to a lack of response to antidepressant treatment. The effects of childhood trauma on increased cortisol levels are specifically evident in patients with glucocorticoid resistance and suggest glucocorticoid resistance as a target for the development of personalized treatment for a subgroup of depressed patients with a history of childhood trauma rather than for all patients with resistance to antidepressant treatment.

## Highlights

• The severity of childhood trauma experience contributes to lack of response to antidepressant treatment.
• Untreated depressed patients show increased diurnal cortisol levels compared with those on antidepressant medication both treatment responder and non-responder patients.
• The severity of childhood trauma contributes to increased HPA axis activity and higher cortisol production specifically in depressed patients who present glucocorticoid resistance.
• Glucocorticoid resistance may be a novel target for development of personalised treatment for a subgroup of depressed patients with a history of childhood trauma.

## 1 Introduction

Major depressive disorder (MDD) is a debilitating condition and the most common psychiatric disorder with more than 300 million people affected worldwide according to the World Health Organisation. Although there are effective antidepressants available, more than one third of depressed patients do not respond to conventional treatments (Cleare et al., 2015, Ferrari et al., 2013). Ongoing studies aim to improve our understanding of the biological mechanisms underlying the causality, development and pathogenesis of depression in order to develop new, more successful and personalized treatment strategies. With growing advances in psychoneuroimmunology research, extensive evidence suggests the importance of abnormal brain-endocrine-immune interaction and dysregulated neuroendocrine and neuroimmune response in the pathogenesis of depression (Miller and Raison, 2016). As shown by the findings from several large population samples and clinical meta-analyses, a significant proportion of depressed patients exhibit hyperactivation of the hypothalamic–pituitaryadrenal (HPA) axis thus increased secretion of the stress hormone cortisol as well as elevated levels of inflammatory biomarkers (Haapakoski et al., 2015, Howren et al., 2009, Perrin et al., 2019, Valkanova et al., 2013, Zunszain et al., 2011). It has been suggested that such disturbances are due to the dysfunction of glucocorticoid receptors (GR), and therefore an impairment in cortisol to mediate HPA axis negative feedback and regulate its own production, and to promote anti-inflammatory response. Indeed, attenuated glucocorticoid responsiveness, so called glucocorticoid resistance, has been consistently observed in MDD patients presenting coexistence of hypercortisolemia and inflammation (Pariante, 2017, Perrin et al., 2019, Stewart et al., 2009).

Early-life traumatic experiences including childhood physical, sexual and emotional abuse and/or neglect are among the most potent contributing risk factors for increased diathesis for major depression in adulthood (Chapman et al., 2004, Heim et al., 2010). Early exposure to life stress has been linked to increased inflammation (Baldwin et al., 2018, Baumeister et al., 2016, Danese et al., 2008) and HPA axis abnormalities such as a dysregulation of the glucocorticoid-mediated negative feedback and an increased cortisol reactivity in adulthood (Harkness et al., 2011, Lu et al., 2016). These neuroendocrine and neuroimmune alterations secondary to childhood traumatic experiences significantly increase vulnerability to the lifetime stress reactivity and subsequent risk for the onset of depressive episodes (Heim et al., 2008, Nemeroff and Binder, 2014).

The heterogeneous nature of MDD with multifactorial aetiological and pathophysiological mechanisms involved, makes treatment more challenging. To date, relatively few investigations have been trying to address this issue and proposing predictors of treatment response in depression. Childhood trauma and maltreatment has been previously associated with poor treatment response to antidepressant pharmacotherapies (Kaplan and Klinetob, 2000, Nanni et al., 2012, Williams et al., 2016). The findings supporting the association between HPA axis hyperactivity and lack of response to treatment in depression are less consistent. While some studies reveal that treatment non-responder depression is associated with hypercortisolemia and elevated inflammation in the presence of glucocorticoid resistance (Carvalho et al., 2010, Carvalho et al., 2008) and that depressed patients with higher inflammation are less responsive to standard antidepressant treatments (Carvalho et al., 2013, Kopschina Feltes et al., 2017); a recent meta-analysis does not support hyperactivation of HPA axis as a predictor for antidepressant treatment response (Fischer et al., 2017). No previous study has investigated in the same sample of patients the association between childhood trauma and HPA axis with antidepressant treatment response.

Considering the costs and challenges for patients and society associated with treatment-resistance in depression, it is important to identify specific and reliable biomarkers predictive of antidepressant efficacy as well as to distinguish those subgroups of depressed patients exhibiting physiological abnormalities affecting their therapeutic response in order to develop new strategies to achieve successful clinical treatment of depression. Therefore, the present study aims to investigate 1) whether experience of childhood trauma is associated with lack of response to antidepressant treatment, 2) whether abnormalities in HPA axis activity (assessed as diurnal cortisol, cortisol awakening response, and glucocorticoid resistance) are associated with lack of response to antidepressant treatment, and 3) whether the experience of childhood trauma is associated with specific abnormalities in HPA axis activity in patients with depression.

## 2 Materials and methods

### 2.1 Subjects

The current cross-sectional study was conducted as part of the BIODEP (BIOmarkers in DEPression) Wellcome Trust Neuroinflammation Consortium, NIMA (Neuroimmunology of Mood Disorders and Alzheimer’s disease), an observational non-interventional study (Chamberlain et al., 2019). Volunteers aged 25–50 years were recruited by a network of clinical research centres in the UK including Brighton, Cambridge, Glasgow, King’s College London and Oxford. The study participants consisted of patients with a current diagnosis of MDD according to Diagnostic and Statistical Manual Version 5 (DSM-5) (American Psychiatric Association, 2013) recruited from mental health and primary national health services as well as healthy adults. Patients were categorised by monoaminergic antidepressant (MA) exposure and therapeutic response at the time of the assessment. Healthy controls were sampled to match the patient group for age and sex. The study was approved by Research Ethics Committee (National Research Ethics Service East of England, Cambridge Central, UK) and conducted according to the Declaration of Helsinki. Written informed consent was obtained from all participants prior to performing any study related activity including eligibility screening examination.

### 2.2 Clinical measures

Diagnosis of MDD and other psychiatric disorders were ascertained by Structured Clinical Interview for DSM-5 (Spitzer et al., 1996). The severity of depressive symptoms was assessed using 17-item Hamilton Rating Scale for Depression (HAM-D_17_) (Hamilton, 1960). The exposure to antidepressant medications was established by Antidepressant Treatment Response Questionnaire (Desseilles et al., 2011). Patients were categorised into three subgroups: treatment responder patients who were not depressed (HAM-D <7) after at least 6 weeks of treatment with a monoaminergic antidepressant (DEP-MA+); treatment non-responder depressed patients who were depressed (HAM-D >13) after at least 6 weeks of treatment with one or more monoaminergic antidepressants (DEP+MA+); and untreated depressed patients who were depressed (HAM-D >17) but had not been treated with monoaminergic antidepressants in the previous 6 months (DEP+MA−). Healthy volunteers had no history of depression requiring treatment with either monoaminergic antidepressants or other clinical interventions including psychotherapy (DEP−MA−).

Exclusion criteria were applied for participants with life time bipolar or non-affective psychotic disorder, sufferers from a medical condition associated with systemic and/or CNS inflammation (such as immunological disorders, cardiovascular disorders, malignancies, recent or current infection confirmed by screening interview and further through the results of the cell blood count, brain disorders) and those with concurrent use of any medication likely to compromise the interpretation of immunological data (such as use of corticosteroids, anti-histamines and anti-inflammatory drugs), current substance use disorders for the last 6 months, participants in clinical trial of an investigational drug within the last 12 months, breast feeding or pregnant women.

Following a semi structured clinical interview, demographic characteristics of the participants were documented. Height and weight were measured for body mass index (BMI) calculation. Participants were also assessed for the childhood trauma by means of Childhood Trauma Questionnaire (CTQ) to measure the severity of different types of childhood trauma including Emotional Abuse, Emotional Neglect, Physical Abuse, Physical Neglect and Sexual Abuse (Bernstein et al., 1994). The Beck Depression Inventory II (BDI-II) (Beck et al., 1996) was also completed and followed by collection of fasting peripheral venous blood between 8:00–10:00am. Participants were then issued with the materials and instruction for collecting the saliva samples.

### 2.3 Peripheral C-reactive protein measurements

The nonspecific acute phase reactant, hsCRP was measured as the most reliable biomarker of on-going inflammation implicated in pathophysiology of depression and its response to treatment (Haroon et al., 2018, Miller and Raison, 2016). Recent research has confirmed that hsCRP is a clinically valuable inflammatory marker, both peripheral and central, and can serve as a proxy for cytokines and other inflammatory mediators, which are more difficult to measure in the clinic (Felger et al., 2018, Miller et al., 2017). The CRP analysis has been described in detail in (Chamberlain et al., 2019) but briefly, the blood samples were collected in clot activator containing tubes for measurements of hsCRP. The samples were allowed to coagulate for 30–60 min then centrifuged at 1600 Relative Centrifugal Force (RCF) for 15 min. The serum samples were separated and transported to a central laboratory (Q2 Solutions) where analysed on the day of receipt. Samples were exposed to anti-CRP-antibodies on latex particles, and the increase in light absorption due to complex formation was used to quantify hsCRP levels, using Turbidimetry on Beckman Coulter AU analyzers. Inter and intra-assay co-efficient of variations were <10%. The Clinical Reporting Range (CRR) for the hsCRP assay was 0.2–9999.9 mg/L.

### 2.4 Salivary cortisol measurements

For cortisol measurements, salivary sampling has been the preferred method over blood sampling in clinical studies and research settings as being an easy to employ, inexpensive, non-invasive and stress-free way to collect samples. A high correlation between plasma and salivary cortisol has been reported in studies investigating HPA axis function indicating that salivary cortisol measurements are a reliable marker of HPA axis assessment (Petrowski et al., 2013). Saliva samples were collected from participants according to our previously published procedure (Mondelli et al., 2010, Nikkheslat et al., 2015). Using salivette sampling devices (Sarstedt, Leicester, UK), the self-collection of samples was carried out at home at 6 time points throughout the day; at awakening, 15, 30 and 60 min after awakening, at 12 pm and at 8 pm. Individuals who described problems during sample collection in the self-recorded questionnaire provided, or who did not respect the time-intervals required, were removed from the analysis. The current study includes those participants who completed saliva sample collections accurately and provided adequate amount of sample for measurements of cortisol. Salivary cortisol levels were measured using a commercially available high sensitivity salivary cortisol enzyme immunoassay kit (Salimetrics). SoftMax Pro 4.8 software was used to calculate the cortisol values, following a 4-parameter fit. The analytical sensitivity was set to 0.19 nmol/l. Inter and intra-assay co-efficient of variations ranged 8–10% and 6–10%, respectively. To investigate the activity and responsiveness of the HPA axis, we first compared the mean values at the various time points of salivary cortisol collection; and secondly, we calculated the area under the curve with respect to the increase (AUCi) for the cortisol awakening response using the 4 time points of 0, 15, 30, and 60 min after awakening; and the area under the curve with respect to the ground (AUCg) for the diurnal cortisol using the 3 main points over the whole day: awakening, noon 12 pm and 8 pm. AUCg indicates the total amount of cortisol produced thus represents the HPA axis overall activity during the day. AUCi measures the variation (either positive or negative) in cortisol concentration thus signifies the HPA axis reactivity and response to the stress of awakening. The formulas for the calculations of the AUC were derived from the trapezoidal formula introduced by Pruessner (Pruessner et al., 2003).

Glucocorticoid resistance was defined as coexistence of increased cortisol production and elevated hsCRP levels as a clinical biomarker of inflammation that indicates an impaired ability of cortisol to exert its anti-inflammatory effect. Therefore, individuals exhibiting the evidence for the presence of glucocorticoid resistance were characterised as those who had both high hsCRP >3 mg/L, which is indicative of an increased inflammation based on widely accepted cut-off points (Pearson et al., 2003) and high diurnal cortisol levels, AUCg >50 nmol/L considering overall sample median. Subjects with the hsCRP values more than 10 mg/L representing the presence of acute inflammation were excluded from the analysis.

### 2.5 Data analysis

All statistical analyses were performed using SPSS software version 24.0. All data were tested for suitability for parametric or non-parametric analysis. For comparisons of the four study groups, including the three groups of MDD patients and the group of controls, we performed one-way ANOVA analysis or Kruskal-Wallis test, where the data violated parametric assumptions confirmed by Shapiro-Wilk test. Tukey’s HSD test was used for post-hoc analysis for the pairwise group differences. Cohen’s d was reported for the effect size of hsCRP, CTQ and cortisol in each clinical group compared to healthy controls. Dichotomous variables including gender and glucocorticoid resistance were compared using Chi-square test. Correlations were assessed using Pearson’s product moment correlation. Mediation analysis was performed using the PROCESS macro. Linear Regression was carried out to investigate the association between childhood trauma and diurnal cortisol in both glucocorticoid resistance and non-resistance groups. A two-way ANOVA with repeated measures was used to determine the differences in salivary cortisol at different time points of the awakening response and during the day among the four groups. General linear model (ANCOVA) and hierarchical multiple regression were performed for taking into account the effect of covariates including BMI, age and gender on the outcomes. The p-values of <0.05 were considered as significant.

## 3 Results

### 3.1 Sample characteristics

A total of 218 participants who provided saliva samples for cortisol measurements were included in this study. The sociodemographic and clinical characteristics of the study sample is presented in Table 1. Treatment responder, treatment non-responder and untreated depressed patients and controls showed similar age range (One-way ANOVA, F = 1.05, p > 0.05) and distribution of gender among the groups (Chi-square, χ^2^ = 0.15, p > 0.05). HAM-D scores were significantly different between each group compared with other groups, as confirmed by the pairwise post hoc analysis (Table 1) (One-way ANOVA, F = 642.01, p < 0.001). BDI-II was positively correlated with the HAM-D_17_ (Pearson, r = 0.757, p < 0.0001). Overall, patients and controls differed significantly on their BMI (One-way ANOVA, F = 2.96, p < 0.05); however, running the post hoc analysis, the difference was only found between treatment responders and controls (p = 0.047). Compared with healthy adults, depressed groups exhibited an increased level of inflammation as indicated by CRP marker (Table 1) (One-way ANOVA, F = 3.9, p < 0.01). Patients also appeared to have experienced greater childhood trauma when each group was compared with controls (Table 1) (One-way ANOVA, F = 12.62, p < 0.001).

**Table 1:** Characteristics of patients with MDD defined by antidepressant exposure and therapeutic response.

	Treatment Responder (1) (n = 42)	Treatment Non-responder (2) (n = 80)	Untreated Depressed (3) (n = 41)	Healthy Control (4) (n = 55)	Test and Significance *Post-hoc Analysis
Age, years (±SD)	36 (±7.8)	36.8 (±7.7)	35.6 (±8.5)	34.5 (±7.2)	F = 1.05, p = 0.37
(95% CI)	(33.5–38.4)	(35.1–38.6)	(32.8–38.2)	(32.5–36.4)	
Gender, female n (%)	29 (69.0%)	57 (71.3%)	28 (68.3%)	38 (69.1%)	χ^2^ = 0.15, p = 0.99
BMI (kg/m2) (±SD)	27.7 (±5.2)	27.1 (±6.9)	25.8 (±4.5)	24.6 (±4.8)	F = 2.96, p = 0.03
(95% CI)	(26.1–29.3)	(25.5–28.6)	(24.3–27.2)	(23.3–26.0)	*1 > 4
HAM-D_17_ score (±SD)	3.6 (±2.9)	18.1 (±3.8)	20.3 (±3.2)	0.7 (±1.1)	F = 642.01, p < 0.001
(95% CI)	(3.0–4.2)	(17.2–18.9)	(19.3–21.3)	(0.4–1.0)	*1, 2, 3 > 4; 2, 3 > 1; 3 > 2
BDI-II score (±SD)	10.7 (±9.8)	26.5 (±10.9)	23.8 (±9.3)	2.3 (±4.1)	F = 91.53, p < 0.001
(95% CI)	(7.6–13.7)	(24.1–28.9)	(20.9–26.7)	(1.2–3.4)	*1, 2, 3 > 4; 2, 3 > 1
CRP (mg/L) (±SD)	2.4 (±2.5)	2.2 (±2.4)	2.0 (±2.3)	1.1 (±1.1)	F = 3.91, p = 0.009
(95% CI)	(1.6–3.2)	(1.7–2.8)	(1.2–2.7)	(0.8–1.4)	*1, 2 > 4
ES Cohen’s d vs HC	0.65	0.58	0.45		
SC (nmol/L)					
SC Awakening (±SD)	9.2 (±4.1)	9.7 (±7.5)	9.1 (±4.7)	9.6 (±5.8)	F = 0.16, p = 0.93
(95% CI)	(7.9–10.5)	(8.1–11.4)	(7.6–10.5)	(8.1–11.2)	
SC 15 min (±SD)	10.4 (±4.6)	11.8 (±7.1)	11.3 (±5.6)		F = 0.61, p = 0.61
(95% CI)	(8.9–11.8)	(10.3–13.5)	(9.5–13.0)	(10.2–13.2)	
SC 30 min (±SD)	10.2 (±4.8)	11.8 (±6.4)	12.3 (±6.4)	12.0 (±5.7)	F = 1.10, p = 0.35
(95% CI)	(8.7–11.7)	(10.4–13.2)	(10.3–14.3)	(10.5–13.6)	
SC 60 min (±SD)	7.4 (±3.7)	8.9 (±6.0)	9.6 (±5.3)	9.9 (±6.2)	F = 1.82, p = 0.14
(95% CI)	(6.2–8.5)	(7.6–10.3)	(7.9–11.3)	(8.2–11.6)	
SC 12 noon (±SD)	4.1 (±2.8)	4.3 (±3.8)	5.4 (±2.9)	4.5 (±3.0)	F = 1.43, p = 0.24
(95% CI)	(3.2–4.9)	(3.5–5.1)	(4.5–6.3)	(3.7–5.4)	
SC 8 pm (±SD)	2.2 (±2.9)	2.2 (±3.3)	2.2 (±3.0)	2.1 (±1.6)	F = 0.04, p = 0.99
(95% CI)	(1.3–3.1)	(1.5–3.0)	(1.3–3.2)	(1.6–2.5)	
AUCi (nmol·min/L)	12.5(±178.9)	65.1 (±211.2)	113.6 (±323.5)	80.06 (±295.9)	H = 2.83, p = 0.42
(±SD) (95% CI)	(−43.2 to 68.2)	(17.8–112.5)	(11.5–215.7)	(−1.5 to 161.6)	
AUCg (nmol·h/L)	53.2 (28.9)	54.4 (38.9)	60.8 (25.2)	59.3 (26.5)	H = 8.72, p = 0.03
(±SD) (95% CI)	(44.2–62.2)	(45.7–63.1)	(52.9–68.8)	(52.0–66.6)	*3 > 1,2
CTQ score Total (±SD)	42.6 (±19.1)	47.8 (±16.9)	53.0 (±16.5)	34.2 (±11.5)	F = 12.62, p < 0.001
(95% CI)	(36.7–48.6)	(44.0–51.5)	(47.8–58.2)	(31.0–37.3)	
ES Cohen’s d vs HC	0.54	0.94	1.32		*1, 2, 3 > 4; 2, 3 > 1
Emotional Abuse (±SD)	9.6 (±5.0)	11.9 (±5.4)	13.6 (±5.4)	6.9 (±2.8)	F = 18.97, p < 0.001
(95% CI)	(8.1–11.2)	(10.7–13.1)	(11.9–15.3)	(6.1–7.6)	
Emotional Neglect (±SD)	11.5 (±5.0)	14.2 (±5.3)	15.5 (±5.0)	9.1 (±3.5)	F = 18.29, p < 0.001
(95% CI)	(9.9–13.0)	(13.0–15.3)	(13.9–17.1)	(8.2–10.1)	
Physical Abuse (±SD)	6.8 (±3.6)	7.0 (±3.5)	7.4 (±3.3)	6.2 (±5.0)	F = 1.09, p = 0.36
(95% CI)	(5.7–7.9)	(6.3–7.8)	(6.4–8.4)	(5.3–7.1)	
Physical Neglect (±SD)	7.8 (±3.7)	7.9 (±3.4)	8.7 (±3.0)	6.4 (±2.5)	F = 4.69, p = 0.004
(95% CI)	(6.6–8.9)	(7.1–8.6)	(7.8–9.6)	(5.7–7.0)	
Sexual Abuse (±SD)	6.9 (±5.0)	6.8 (±4.4)	7.8 (±5.7)	5.6 (±2.4)	F = 1.96, p = 0.12
(95% CI)	(5.4–8.5)	(5.9–7.8)	(6.0–9.6)	(5.0–6.3)	

AUCg = Area Under the Curve ground, AUCi = Area Under the Curve increase, BDI = Beck depression inventory, BMI = body mass index, CRP = C-reactive protein, CTQ = Childhood Trauma Questionnaire, HAM-D_17_ = Hamilton Depression Rating Scale 17 for Depression, ES = Effect Size, HC = Healthy Control, SC = Salivary Cortisol

* Tukey’s HSD pairwise group significant differences (p < 0.05).

### 3.2 Childhood trauma, depression and antidepressant response

Depressed individuals had significantly higher exposure to childhood trauma when compared with healthy adults (U = 2089.50, z = −5.919, p < 0.0001), and this was also true when each subgroup of patients was compared with controls (Table 1). Comparing the two groups of patients under medication, treatment non-responder depressed patients revealed significantly higher CTQ total scores than treatment responders (U = 1261.00, z = −2.259, p = 0.024). Examining the potential mediating role of cortisol and hsCRP on the association between childhood trauma and adulthood depressive symptoms in treatment non-responder depressed patients indicated that the association was not mediated by the effect of cortisol (Direct effect of CTQ on BDI-II: B = 0.285, p = 0.0038 and indirect effect via AUCg: p > 0.05, CI −0.294, 0.014; direct effect of CTQ on AUCg: B = 0.0007, p = 0.998, and AUCg on BDI-II: B = −0.024, p = 0.423) or hsCRP (Direct effect of CTQ on BDI-II: B = 0.204, p = 0.0048 and indirect effect via hsCRP: p > 0.05, CI −0.031, 0.026; direct effect of CTQ on hsCRP: B = 0.012, p = 0.459, and hsCRP on BDI-II: B = 0.398, p = 0.417) in these patients.

### 3.3 Cortisol production and glucocorticoid resistance

**Fig. 1:**
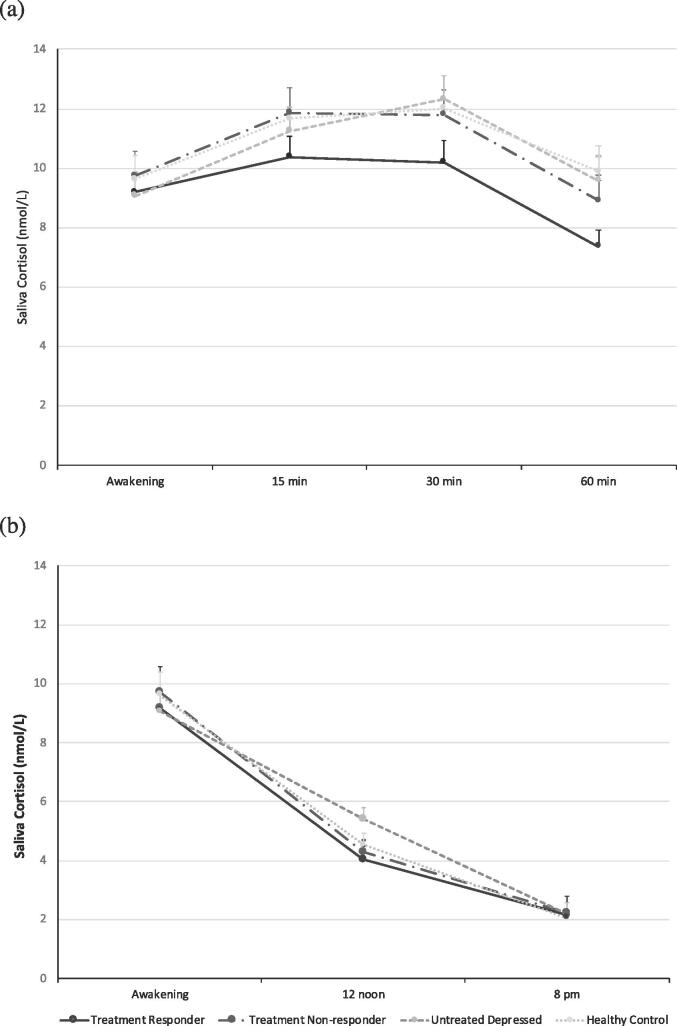
Mean salivary cortisol levels for the cortisol awakening response (a) and diurnal cortisol secretion (b) in patients with depression (treatment responder, treatment non-responder and untreated depressed groups) and healthy subjects (Two-way ANOVA, p > 0.05).

The repeated measures ANOVA comparing the salivary cortisol at different time points among the groups showed no significant differences in the levels of cortisol at each time point between three groups of patients with depression (treatment responder, treatment non-responder and untreated patients) and controls, for both the first hour post awakening (Fig. 1a) (Two-way ANOVA for the between-group effect, F(3,3) = 0.793, p = 0.50), and during the day (Fig. 1b) (Two-way ANOVA for the between-group effect, F(3,2) = 0.137, p = 0.94). The results from calculating the AUCi (nmol·min/L) for the cortisol awakening response (Fig. 1a) by measuring the variation (either positive or negative) in cortisol concentration revealed no differences in the HPA axis reactivity and response to the stress of awakening among the groups (Kruskal-Wallis, H = 2.83, p = 0.42) (Table 1).

The AUCg for the diurnal cortisol (nmol·hr/L) (Fig. 1b), indicative of the HPA axis overall activity by measuring the total amount of cortisol concentrations, was significantly different among the groups (Kruskal-Wallis, H = 8.721, p = 0.033); the post-hoc analyses revealed significant difference between untreated depressed and treatment responder (U = 630.00, z = −2.104, p = 0.035, ES Cohen’s d = 0.28) and between untreated depressed and treatment non-responder patients (U = 1192.50, z = −2.451, p = 0.014, ES Cohen’s d = 0.2) (Table 1). Indeed, combining the two groups of patients who were on medication (both treatment responder and treatment non-responder) showed significantly lower diurnal cortisol production when compared with untreated depressed individuals (U = 1822.50, z = −2.595, p = 0.009).

**Fig. 2:**
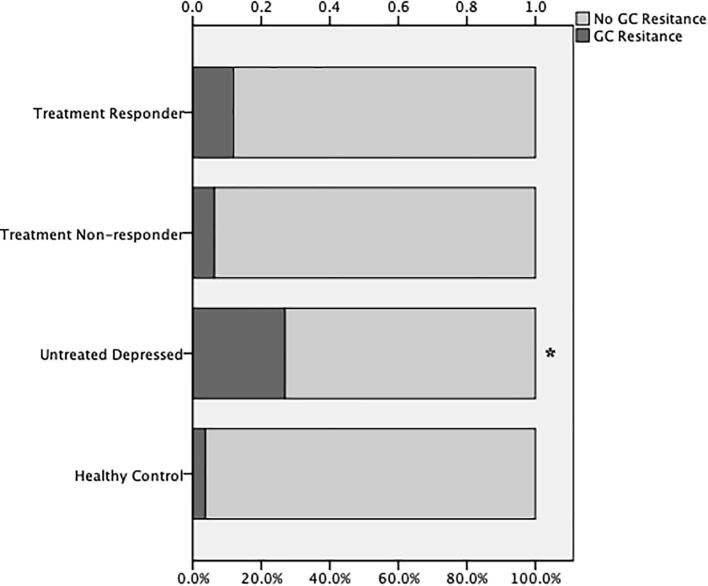
Patients with depression who were untreated had a significantly higher proportion of individuals with glucocorticoid (GC) resistance (hypercortisolemia and high CRP) compared with medicated patients with depression (both treatment responder and treatment non-responder) as well as with controls (χ^2^ = 15.948, p < 0.01).

Investigating the presence of glucocorticoid resistance, MDD patients showed higher prevalence of glucocorticoid resistance as compared with healthy adults (Chi-square, χ^2^ = 3.726, p = 0.05). Further analysis revealed that depressed patients who were untreated had significantly higher proportion of individuals (26.8%) with glucocorticoid resistance (diurnal cortisol secretion above median and hsCRP >3 mg/L), than both treatment responder (11.9%) and treatment non-responder patients (6.3%), as well as controls (3.6%) (Chi-square, χ^2^ = 15.948, p = 0.001) (Fig. 2).

### 3.4 Association of childhood trauma with cortisol and glucocorticoid resistance

The severity of childhood trauma was not found to be associated with higher cortisol levels in depressed patients (p > 0.05). However, in individuals with glucocorticoid resistance, childhood trauma was positively associated with greater diurnal cortisol output in adulthood (F = 34.775, p < 0.0001), as opposed to individuals with no glucocorticoid resistance (F = 1.278, p = 0.26) (Table 2).

**Table 2:** Linear regression for association between childhood trauma (CTQ total score) and diurnal cortisol production (AUCg of diurnal cortisol nmol·h/L) in individuals with glucocorticoid resistance compared with those with no glucocorticoid resistance.

	R	R^2^	Adjusted R^2^	F	95% CI	B	Beta	p value
GC Non-Resistance	0.082	0.007	0.001	1.278	−0.436 to 0.118	−0.159	−0.082	0.260
GC Resistance	0.790	0.623	0.606	34.775	0.521–1.088	0.805	0.790	**<0.0001**

### 3.5 Covariate analysis

The effect of BMI as a confounder has been analysed on serum hsCRP, cortisol awakening response, diurnal salivary cortisol and the statistically significant results remained unchanged. The regression model has been also controlled for age, gender and BMI and the outcome remained unchanged.

## 4 Discussion

Our results show that depressed individuals, regardless of treatment response, had more experience of childhood trauma than healthy subjects. When considering the response to antidepressant medications, treatment non-responder patients had higher exposure to childhood trauma compared with responders. We did not find any specific HPA axis activity abnormality to be associated with non-response to the treatment. However, depressed patients who were on antidepressant medication showed lower total diurnal cortisol production compared with untreated patients. In addition, we found that the untreated group had higher proportion of individuals presenting with glucocorticoid resistance than medicated depressed patients and controls. Severity of childhood trauma was associated with increased diurnal cortisol levels only in individuals with glucocorticoid resistance.

Cortisol is the primary end-product and the main peripheral marker of the HPA axis. Studies reveal mixed results when looking at the link between depression and the HPA axis as a principal endocrinological stress response system, with most findings suggesting the presence of HPA axis hyperactivation characterised by increased cortisol concentrations in almost half of MDD patients (Pariante, 2017, Perrin et al., 2019, Herbert, 2013, Vreeburg et al., 2009). However, a systematic review and meta-analysis detected only a small difference in salivary cortisol levels between depressed patients and controls, and recommended the interpretation of results with caution due to a substantial overlap between values in the two groups, presence of random errors and bias, and large heterogeneity between studies (Knorr et al., 2010). According to another meta-analysis, the degree of hypercortisolemia in depression may vary considerably depending on the type of depression and the age of patients included in the studies. The authors concluded that greater cortisol differences between depressed and non-depressed were mostly reported in older patients and also more in hospitalised individuals than outpatients (Stetler and Miller, 2011). These outcomes may explain no differences in cortisol awakening response and diurnal cortisol levels among the groups (Fig. 1) of our current study, which was mainly focussing on a population of younger outpatient adults. These findings further suggest the HPA axis abnormalities may be more pronounced in specific groups of patients or may reflect more severe depressive states (hospitalized patients).

The investigation on the association between antidepressant treatment and HPA axis activity by means of measuring AUCg for the diurnal cortisol output revealed lower cortisol levels in medicated depressed patients, both treatment responder and treatment non-responder, than those who were untreated. It appears that antidepressant medications, rather than depression status and regardless to the responsiveness, influence cortisol production in patients with depression. A recent meta-analysis assessed HPA axis functioning as predictor of depressive response and reported no differences in cortisol secretion between treatment responders and non-responders. Following the meta-regression analysis, the study revealed that compared with responders, the hypercortisolemia in non-responders were demonstrated only in those studies with no report on sample handling, no account for controlling for age, and excluding comorbidities (Fischer et al., 2017). While altered HPA axis alone may not be a robust marker for predicting treatment response, the findings supporting the effect of antidepressants in normalizing HPA axis dysregulation in depression cannot be overlooked (Pariante, 2017, Pariante and Lightman, 2008). Indeed, it has been evident that antidepressants, apart from their classic mechanism of action on the neurotransmitter system, have a distinct role in normalising HPA axis abnormalities by directly enhancing GR function and expression, thus promoting cortisol-mediated negative feedback regulation of the axis (Anacker et al., 2011a, Nikkheslat et al., 2017). The mechanism of antidepressants action on GR and activation of glucocorticoid molecular pathways in various target tissues provides also other putative therapeutic related effects for example, by increasing hippocampal neurogenesis and reducing peripheral inflammation (Anacker et al., 2011b, Anacker et al., 2013, Carvalho et al., 2010).

Our results revealed that depressed individuals were more likely to exhibit evidence of glucocorticoid resistance (coexistence of high cortisol and high hsCRP), and that untreated patients specifically had greater proportion of individuals presenting glucocorticoid resistance than medicated depressed patients and controls (Fig. 2). Immune activation and inflammation have been consistently reported in depressed patients (Dowlati et al., 2010, Haapakoski et al., 2015, Huang et al., 2019, Valkanova et al., 2013). Regulation of inflammatory response is one of the key roles of glucocorticoids. The notion of glucocorticoid resistance in relation to inflammation has been studied extensively over the last two decades (Pariante, 2017). Insights into molecular mechanisms underlying inflammatory dysregulation in depression disclosed a bidirectional association between inflammation and GR. Prolonged inflammation and activated cytokine-signalling pathway interact with GR-signalling pathway leading to a decrease in GR sensitivity. A dysfunctional GR in turn shows an impaired responsiveness to cortisol and a reduced ability to perform anti-inflammatory action (Pace et al., 2007, Zunszain et al., 2011). Interestingly, it has been demonstrated that some antidepressants are able to improve GR function (Carvalho et al., 2010, Carvalho and Pariante, 2008, Pariante et al., 1997) and reverse glucocorticoid resistance (Pariante et al., 2012, Pariante et al., 2004). The findings, which support our result showing less prevalence of glucocorticoid resistance in medicated depressed groups, both treatment responder and treatment non-responder, suggest that antidepressant drugs, regardless of whether depressive symptoms persist, may exert anti-inflammatory effects through improving the effectiveness of cortisol response.

Studies on treatment resistance depression also demonstrated elevated inflammatory response in the context of HPA axis hyperactivation and hypercortisolemia in severely depressed non-responder inpatients (Carvalho et al., 2008). The presence of glucocorticoid resistance was further confirmed on the same group of patients by *in vitro* evaluation of GR on their peripheral blood mononuclear cells that were unable to respond to synthetic glucocorticoid, dexamethasone, during immune challenge by bacterial lipopolysaccharide (Carvalho et al., 2010). Following the evidence suggesting the association of inflammation with depression and lack of clinical therapeutic benefit of antidepressants (Carvalho et al., 2013), recent research is focusing on targeting inflammation and use of anti-inflammatory medications in treatment of depression for those subgroups of patients who may require alternative or adjuvant therapeutic strategies (Husain et al., 2017, Kopschina Feltes et al., 2017, Miller and Raison, 2016). In our study, although the treatment non-responder group exhibit elevated inflammation and presence of glucocorticoid resistance in a subset of patients, we have not found significantly higher levels of hsCRP, cortisol and GR resistance compared with treatment responders; this finding would suggest involvement of other pathophysiological mechanisms and aetiological factors associated with their response to treatment, that is important to take into account, considering the heterogeneous nature of MDD.

Childhood maltreatment as one of the most potent risk factors for development of depression adversely affects the course of illness and response to the antidepressant treatment (Kaplan and Klinetob, 2000, Williams et al., 2016). A meta-analysis looking at both epidemiological and clinical studies revealed that childhood maltreatment follows a greater risk of developing both recurrent and persistent depressive episodes in depressed adults and with poorer treatment outcome compared with depressed individuals without a history of childhood maltreatment (Nanni et al., 2012). Testing the hypothesis on the association between childhood trauma, depression and treatment response in line with mounting evidence documented up to date, the current study population of depressed patients have also shown more experience of childhood trauma compared with healthy controls. When the response to antidepressant medications was considered, treatment non-responder individuals had indeed higher exposure to childhood trauma than treatment responders. In addition, the association between childhood trauma and adulthood depressive symptoms in treatment non-responder patients was not found to be mediated by the effect of cortisol or hsCRP. These findings highlight the importance of early preventive and therapeutic interventions as well as enquiring information about childhood trauma as a routine clinical assessment of depression in order to identify individuals at higher risk of poor response to treatment. However, establishing alternative and more effective treatment options requires comprehensive understanding of the specific physiological phenotype and targeting those responsible biological abnormalities in this depressed group. It is important to note that not all individuals who are exposed to trauma in their childhood would develop depression later in life and some even demonstrate resilience to depression when exposed to additional stressors throughout life; this suggests the presence of opposite biological responses across different individuals probably mediated by genetic and epigenetic factors (Heim et al., 2009, Monroe and Reid, 2008).

Recently, more research has attempted to identify the pathophysiological mechanisms underlying development of depression triggered by early life stress and to establish the existence of biologically distinguishable subtypes of depression associated with childhood trauma and to predict individuals who would be responsive to treatment (Harkness et al., 2011, Heim et al., 2008, Lu et al., 2016). Indeed, early exposure to life stress has been linked to neuroendocrine and neuroimmune changes including alterations in HPA axis activity (Harkness et al., 2011, Lu et al., 2016), increased cortisol reactivity and pro-inflammatory state in adulthood (Baumeister et al., 2016, Danese et al., 2007). In our attempt to investigate HPA axis function and inflammation as underlying physiological mechanisms linking childhood trauma with depression and treatment response, we have found that (Table 2) the severity of childhood trauma was associated with higher cortisol levels only in individuals exhibiting glucocorticoid resistance but not in those without glucocorticoid resistance or the overall depressed subjects. These findings indicate different neuroendocrine and neuroimmune responses to childhood trauma in individuals with depression. Based on evidence suggesting that neuroendocrine and neuroimmune alterations secondary to childhood trauma significantly increase vulnerability to the lifetime stress reactivity and subsequent risk for the onset of depressive episodes (Heim et al., 2008, Nemeroff and Binder, 2014), our findings suggest that development of depression in adults exposed to early life trauma, is associated with increased HPA axis activation in the context of glucocorticoid resistance. Coexistence of hypercortisolemia and inflammatory biomarkers represents insufficient glucocorticoid signalling in this subgroup of patients probably due to impairment in GR functioning. Direct evaluation of GR functional properties has been extensively studied in depression and in relation to antidepressants treatment. With regards to early life stress and future development of depression, there is evidence from genetic studies on the association of polymorphisms in genes regulating the HPA axis and GR with depression and childhood trauma (Bet et al., 2009, Heim and Binder, 2012, Roy et al., 2010). Epigenetic studies also have shown a decrease hippocampal GR expression in animal models (Meaney and Szyf, 2005) and in abused suicide victims (McGowan et al., 2009).

To our knowledge this study is the first to investigate measures of HPA axis and inflammation in the context of glucocorticoid resistance in relation to childhood trauma, depression and treatment response in the same population. The large sample size confirms validity of findings from other smaller studies, which also investigated only some of these pathophysiological disturbances. Despite a comprehensive assessment, there are few limitations need to be mentioned. Firstly, patients were not stratified according to depression subtypes. Based on the evidence atypical depression may not be associated with HPA axis hyperactivity and hypercortisolemia, which are observed in melancholic depressive subtype (Juruena et al., 2018). In addition, since this is a cross-sectional study, future longitudinal studies are needed to confirm our findings. Finally, we only measured indirectly glucocorticoid resistance, where high cortisol and high CRP provide the evidence for glucocorticoid resistance, and therefore, an evaluation of GR function both by *in vivo* assessment on the HPA axis (dexamethasone suppression test) and *in vitro* measurements on blood immune cells (dexamethasone-inhibition of LPS-induced cytokine levels) in future studies are needed to confirm an impairment of the GR-mediated glucocorticoid resistance. It would be also valuable for future studies to evaluate other inflammatory markers which have been associated with both childhood trauma and depression, as well to investigate potential differential effects of antidepressant on immune processes.

## 5 Conclusion

Although we found a link between childhood trauma and non-response to the antidepressant treatment in our depressed patients, we did not find specific evidence of HPA axis hyperactivity or glucocorticoid resistance in our treatment non-responder patients. However, our results showed that untreated depressed patients had increased diurnal cortisol and a higher prevalence of glucocorticoid resistance when compared with depressed patients on antidepressant medication. Our study shows that severity of childhood trauma contributes to increased HPA axis hyperactivity specifically in patients who present glucocorticoid resistance. These findings support a role of glucocorticoid resistance in amplifying the effects of childhood trauma in activating the HPA axis and suggest glucocorticoid resistance as a target for the development of personalised treatment for a subgroup of depressed patients with a history of childhood trauma rather than for all patients with resistance to antidepressant treatment. Future studies should investigate alternative or adjuvant treatment strategies that may improve glucocorticoid signalling in affected individuals and thereby reduce both inflammation and depressive symptoms.

## Funding and disclosure

This work was funded by a grant from the 10.13039/100010269Wellcome Trust (Grant number: 104025/Z/14/Z) to the NIMA Consortium, which is also funded by Janssen, 10.13039/100004330GlaxoSmithKline, 10.13039/501100013327Lundbeck and 10.13039/100004319Pfizer. Recruitment of patients was supported by the National Institute of Health Research (10.13039/501100000272NIHR) Clinical Research Network: Kent, Surrey and Sussex & Eastern. The work is also supported by the 10.13039/501100000265Medical Research Council (UK) MR/J002739/1 and the 10.13039/100011102Commission of European Communities Seventh Framework Programme (Collaborative Project Grant Agreement no. 22963, Mood Inflame); and part funded by the NIHR/Wellcome Trust, King's Clinical Research Facility and the NIHR Biomedical Research Centre [and Dementia Unit] at South London and Maudsley NHS Foundation Trust and King’s College London. Dr Valeria Mondelli is supported by MQ: Transforming Mental Health (Grant: MQBF1) and by the Medical Research Foundation (Grant: MRF-160-0005).

SRC consults for Cambridge Cognition and Shire; and his input in this project was funded by a 10.13039/100010269Wellcome Trust Clinical Fellowship (110049/Z/15/Z). ETB is employed half-time by the University of Cambridge and half-time by GlaxoSmithKline; he was holding stock (not currently) in GSK. NAH consults for GSK. PdB, DJ and WCD are employees of Janssen Research & Development, LLC., of Johnson & Johnson, and hold stock in Johnson & Johnson.

## Declaration of Competing Interest

The authors declare that they have no known competing financial interests or personal relationships that could have appeared to influence the work reported in this paper.
