# Supplementary material for: Childhood trauma, HPA axis activity and antidepressant response in patients with depression
Source: Brain Behav Immun. 2020 Jul;87:229–37. doi: 10.1016/j.bbi.2019.11.024 (PMC7327513; doi:10.1016/j.bbi.2019.11.024)
Supplement: Supplementary data 2 [file mmc2.docx]

**Annex: NIMA (Part 1) Consortium members**

Cambridge

Edward T. Bullmore (MD, PI, EC)^1,2,11^, Junaid Bhatti^1^, Samuel J. Chamberlain^1,2^, Marta M. Correia^1,12^, Anna L. Crofts^1^, Amber Dickinson*, Andrew C. Foster*, Manfred G. Kitzbichler^1^, Clare Knight*, Mary-Ellen Lynall^1^, Christina Maurice^1^, Ciara O'Donnell^1^, Linda J. Pointon^1^, Peter St George Hyslop^1,13,14^, Lorinda Turner^31^, Petra Vertes^1^, Barry Widmer^1^, Guy B. Williams^1,14^

Cardiff

B. Paul Morgan (PI)^15^, Claire A. Leckey^15^, Angharad R. Morgan*, Caroline O'Hagan*, Samuel Touchard^15^

Glasgow

Jonathan Cavanagh (PI, EC)^3^, Catherine Deith*, Scott Farmer^16^, John McClean^16^, Alison McColl^3^, Andrew McPherson*, Paul Scouller*, Murray Sutherland^16^

Independent advisor

H.W.G.M. (Erik) Boddeke (EC)^17^

GSK

Jill C. Richardson (EC)^18^, Shahid Khan^11^, Phil Murphy^19^, Christine A. Parker^19^, Jai Patel^11^

Janssen

Declan Jones (EC)^6^, Peter de Boer^4^, John Kemp^4^, Wayne C. Drevets^6^, Jeffrey S. Nye (deceased), Gayle Wittenberg^6^, John Isaac^6^, Anindya Bhattacharya^6^, Nick Carruthers^6^, Hartmuth Kolb^6^

Kings College London

Carmine M. Pariante (PI)^10^, Federico Turkheimer (PI)^20^, Gareth J. Barker^20^, Heidi Byrom^10^, Diana Cash^20^, Annamaria Cattaneo^10^, Antony Gee^20^, Caitlin Hastings^10^, Nicole Mariani^10^, Anna McLaughlin^10^, Valeria Mondelli^10^, Maria Nettis^10^, Naghmeh Nikkheslat^10^, Karen Randall^20^, Hannah Sheridan*, Camilla Simmons^20^, Nisha Singh^20^, Victoria Van Loo*, Marta Vicente-Rodriguez^20^, Tobias C. Wood^20^, Courtney Worrell*, Zuzanna Zajkowska*

Lundbeck

Niels Plath (EC)^21^, Jan Egebjerg^21^, Hans Eriksson^21^, Francois Gastambide^21^, Karen Husted Adams^21^, Ross Jeggo*, Christian Thomsen^21^, Jan Torleif Pederson^21^, Brian Campbell*, Thomas Möller*, Bob Nelson*, Stevin Zorn*

University of Texas (sub-contracted to Lundbeck)

Jason O'Connor^22^

Oxford

Mary Jane Attenburrow (PI)^7,23^, Alison Baird, Jithen Benjamin^23^, Stuart Clare^25^, Philip Cowen^7^, I-Shu (Dante) Huang^24^, Samuel Hurley*, Helen Jones^23^, Simon Lovestone^7^,(AD, PI, EC) Francisca Mada*, Alejo Nevado-Holgado^7^, Akintayo Oladejo*, Elena Ribe^7^, Katy Smith^23^, Anviti Vyas*

Pfizer

Zoe Hughes*, Rita Balice-Gordon*, James Duerr*, Justin R. Piro*, Jonathan Sporn*

Southampton

V. Hugh Perry (PI)^27^, Madeleine Cleal*, Gemma Fryatt^27^, Diego Gomez-Nicola^27^, Renzo Mancuso^32^, Richard Reynolds^27^

Sussex

Neil A. Harrison (PI, EC)^28^, Mara Cercignani^28^, Charlotte L. Clarke^28^, Elizabeth Hoskins*, Charmaine Kohn*, Rosemary Murray*, Lauren Wilcock^29^, Dominika Wlazly^30^

University of Toronto (sub-contracted to Cambridge)

Howard Mount^13^

MD = Mood disorder workpackages lead

AD = Alzheimer’s disease workpackages lead

PI = Principal Investigator

EC = Executive committee member

^1^ Department of Psychiatry, School of Clinical Medicine, University of Cambridge, CB2 0SZ, UK

^2^ Cambridgeshire and Peterborough NHS Foundation Trust, Cambridge, CB21 5EF, UK

^3^ Sackler Centre, Institute of Health & Wellbeing, University of Glasgow, Sir Graeme Davies Building , Glasgow, G12 8TA, UK

^4^ Neuroscience, Janssen Research & Development, Janssen Pharmaceutica NV, Turnhoutseweg 30, B-2340, Beerse, Belgium

^5^ The Maurice Wohl Clinical Neuroscience Institute, Cutcombe Road, London, SE5 9RT, UK

^6^ Neuroscience, Janssen Research & Development, LLC, Titusville, NJ, 08560, USA

^7^ Department of Psychiatry, University of Oxford, Warneford Hospital, Oxford, OX3 7JX, UK

^8^ Brighton & Sussex Medical School, University of Sussex, Brighton, BN1 9RR, UK

^9^ Sussex Partnership NHS Foundation Trust, Swandean, BN13 3EP, UK

^10^ Kings College London, Institute of Psychiatry, Psychology and Neuroscience, Department of Psychological Medicine, London, SE5 9RT, UK

^11^ Immuno-Psychiatry, Immuno-Inflammation Therapeutic Area Unit, GlaxoSmithKline R&D, Stevenage SG1 2NY, UK

^12^ MRC Cognition and Brain Sciences Unit, 15 Chaucer Road, Cambridge CB2 7EF, UK

^13^ Tanz Centre for Research in Neurodegenerative Diseases, 60 Leonard Avenue, Toronto, ON M5T 2S8 Canada

^14^ Department of Clinical Neurosciences, University of Cambridge, CB2 0SZ, UK

^15^ Cardiff University, Cardiff CF10 3AT, UK

^16^ NHS Greater Glasgow and Clyde, 1055 Great Western Rd, Glasgow G12 0XH, UK

^17^ University of Groningen, 9712 CP Groningen, Netherlands

^18^ Neurosciences Virtual PoC DPU, GlaxoSmithKline R&D, Stevenage SG1 2NY, UK

^19^ Experimental Medicine Imaging, GlaxoSmithKline R&D, Stevenage SG1 2NY, UK

^20^ King's College London, Department of Neuroimaging Sciences, Institute of Psychiatry, Psychology & Neuroscience, De Crespigny Park, London SE5 8AF, UK

^21^ H. Lundbeck A/S Ottiliavej 9, 2500, Valby, Denmark

^22^ University of Texas Health Science Center at San Antonio, 7703 Floyd Curl Dr, San Antonio, TX 78229, USA

^23^ NIHR Oxford cognitive health Clinical Research Facility, Warneford Hospital, Oxford, OX3 7JX, UK

^24^ The Kennedy Institute of Rheumatology, Roosevelt Dr, Oxford OX3 7FY, UK

^25^ Oxford Centre for Functional MRI of the Brain, John Radcliffe Hospital, Oxford OX3 9DU, UK

^26^ Pfizer, Inc, 1 Portland Street, Cambridge MA, USA

^27^ Centre for Biological Sciences, University of Southampton, Southampton, UK

^28^ Clinical Imaging Sciences Centre (CISC), University of Sussex, Brighton, BN1 9RR, UK

^29^ Sussex Partnership NHS Foundation Trust, Nevill Avenue, Hove BN3 7HZ, UK

^30^ Brighton & Sussex University Hospitals NHS Trust, Brighton BN2 5BE, UK

^31^ Department of Medicine, School of Clinical Medicine, University of Cambridge, CB2 0SZ, UK

^32^ VIB-KU Leuven Center for Brain & Disease Research, Campus Gasthuisberg, Herestraat 49, bus 602, 3000 Leuven, Belgium

*Former consortium members
